# Somatic comorbidities and Alzheimer’s disease treatment

**DOI:** 10.1007/s10072-013-1290-3

**Published:** 2013-02-01

**Authors:** Alessandra Clodomiro, Pietro Gareri, Gianfranco Puccio, Francesca Frangipane, Roberto Lacava, Alberto Castagna, Valeria Graziella Laura Manfredi, Rosanna Colao, Amalia Cecilia Bruni

**Affiliations:** 1 Regional Neurogenetic Centre, ASP Catanzaro, 88046 Lamezia Terme (CZ), Italy; 2 ASP Catanzaro, 88100 Catanzaro, Italy

**Keywords:** Dementia, Alzheimer’s disease, Drug treatment, Somatic comorbidities, Side effects/adverse events, Elderly

## Abstract

Therapeutic strategies in Alzheimer’s disease (AD) must take into account the characteristics of elderly people, who often have somatic comorbidities. Moreover, demented patients are more frequently frailer than older people. They have a higher number of admissions to hospital, a greater prevalence of complications and an increased risk of death. Therapeutic decisions for these patients have to be approached cautiously: aging, a more elevated comorbidity/polytherapy index and frailty contribute to enhance the risk of pharmacological adverse events and drug interactions. The aim of the present study was to focus on risk–benefit profile of pharmacological therapy for AD in relation to somatic comorbidities that often affect these patients. A Medline search (from 2001 to 2012) was performed using as key words dementia, Alzheimer’s disease, drug treatment, somatic comorbidities, side effects/adverse events and elderly. Cholinesterase inhibitors (ChEIs) and memantine represent the main pharmacological strategies effective in reducing the progression of cognitive decline and functional loss in AD. Many conditions very common in the elderly may restrict the use of ChEIs and/or treatment efficacy in AD patients. Memantine has a good efficacy and tolerability profile with better safety in pulmonary, cardiovascular and central nervous system comorbidities compared to ChEIs. Drug interactions with memantine are also more favorable since they concern mostly drugs not commonly used in the elderly. Only a careful evaluation of the associated somatic diseases, taking into account different drugs safety indexes and tolerability, can lead to personalized treatment management, in order to maximize drug efficacy and optimize quality of life.

## Introduction

The unprecedented extension of life expectancy in western countries is associated with a social and medical burden due to the growing number of chronic diseases. Among older people, aging and coexistence of multiple disease can contribute to create a frail status: this condition is characterized by a reduction in functional reserve in organs and systems that nearly precede symptoms of failure. Frailty correlates with age and represent an independent predictor of death [1].

In 2005, approximately one person in two over 65 years of age in Italy had at least one chronic disease: 34.9 % of men and 47.4 % of women were affected by three or more chronic conditions (ISTAT 2007). Data coming from two population studies performed in Calabria focusing on Frontotemporal dementia [2] and Chronic fatigue syndrome [3] revealed that 93 % of subjects over 65 had at least one chronic and 47.8 % had three or more. Women were sicker than men (51 vs 44 %) (unpublished data). In the Swedish population, 55 % of people over 76 years suffered from at least two chronic diseases, most commonly hypertension (38 %), dementia (21 %), heart failure (18 %) and neurosensorial deficits (about 15 %) [4].

Dementia represents one of the main causes of disability in later life: prevalence rates in community studies increase from 30 % (85–89 years) to 50 % (90–94 years) reaching 74 % for those 95 years or older [5]. Alzheimer’s disease is one of the most common forms of dementia (about 40–50 % of dementia cases), affecting 6–10 % of people over 65 years and doubling every 5 years after age 65 [6]. A global world prevalence of 24 million has recently been calculated [7].

People affected by dementia often present with additional chronic medical conditions (comorbidity): patients attending primary care have on average 2.4 chronic conditions and receive 5.1 medications [8]. Recent studies describe dementia patients as sicker than older people without dementia [9, 10], often showing a specific pattern of concurrent somatic diseases (non-psychiatric), mostly cardiovascular, genitourinary, musculoskeletal and neurological in nature [11]. Moreover, the different stages of dementia seem to be related to different comorbidity patterns. Tumors, diabetes and gastrointestinal diseases are more prevalent in mild to moderate stages, whereas pneumonia, other infectious diseases, stroke, malnutrition, hip fractures and bedsores are the main conditions associated with severe dementia [12]. Recently, an Italian study diagnosed as frail 50 % of AD outpatients, according to the study of osteoporotic fractures (SOF) criteria. Frailty independently correlated with age and loss of autonomy in the basic activities of daily living and was an independent predictor of death at 1 year [13].

**Fig. 1:**
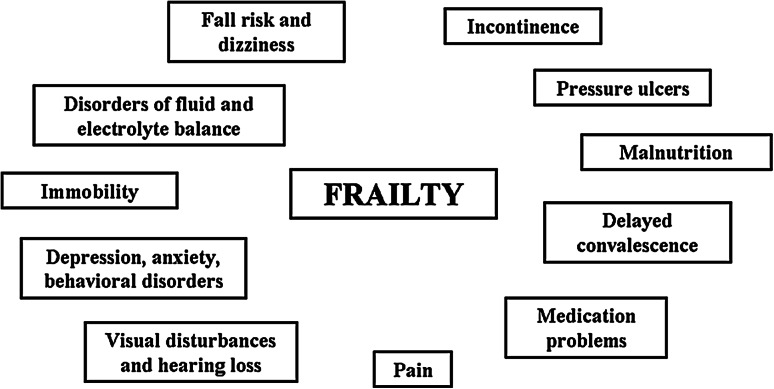
Possible list of symptoms and conditions frequently characterizing patients with dementia

Figure 1 reports the possible list of the symptoms and conditions frequently characterizing patients with dementia.

According to these data, people affected by dementia also have a higher number of admissions to hospital and a higher prevalence of complications such as pneumonia, hip fracture and, secondarily, pulmonary embolism, renal failure, septicemia, urinary infections. Atherosclerosis (32.9 %), hypertension (27.3 %), coronary artery disease (19.5 %), bladder/urethral disorders (13.8 %), congestive heart failure (12.2 %) and ischemic stroke or transient ischemic attack (TIA) (11.5 %) represent the main discharge diagnoses in 4,466 hospitalized older patients with dementia [14]. They also have an increased risk of death during hospitalization [14]. A review of autopsy reports on 52 demented patients showed bronchopneumonia as the most common cause of death (24 cases, 46.1 %) followed by 19 cases of emphysema (36.5 %) and 9 cases of pulmonary thromboembolism (17.3 %, 6 of which were fatal). Twenty-one (40.3 %) of the 52 patients had evidence of myocardial infarction and 38 had atherosclerotic cardiovascular disease. Four clinically unsuspected malignancies were found (glioblastoma multiforme, diffusely infiltrative central nervous system lymphoma, pancreatic adenocarcinoma and adenocarcinoma of the lung) [15].

Therefore, in older people AD itself carries several comorbidities and frailty. Conversely, comorbidities may worsen the clinical course in AD patients (i.e., by accelerating cognitive and functional decline) and often complicate their pharmacological management [11]. Careful surveillance of abrupt changes in clinical status of patients with dementia can usually signal an intercurrent, often treatable, illness or condition (e.g., urinary infections, pneumonia, malnutrition, constipation) [11]. Especially if poorly treated, acute exacerbation of conditions such as congestive heart failure, coronary artery disease, hypertension, diabetes, or chronic obstructive pulmonary disease may adversely affect the cognitive function of patients with dementia; treating congestive heart failure or chronic obstructive pulmonary disease can also relieve dyspnea, pain, agitation and depression [11].

In the last 20 years, many efforts have been made to develop therapeutic strategies able to modify the natural history of Alzheimer’s disease (AD). At present, cholinesterase inhibitors (ChEIs: donepezil, rivastigmine, galantamine) and, most recently, the noncompetitive glutamate *N*-methyl-d-aspartate (NMDA) receptor blocker and adamantine derivative memantine, represent the main pharmacological strategies effective in reducing the progression of cognitive decline and functional loss in people affected by AD.

Prescription of AD therapies must unavoidably take into account the characteristics of older population such as comorbidity, frailty and their complex interrelations. Attention to comorbidities and their treatment are important factors in any AD care plan. Although AD treatment has been revealed to be useful in modulating the disease course, it has been demonstrated that when older AD patients were prescribed several drugs, fewer agents were dedicated to AD [16].

In any case, therapeutic decisions must be not only appropriate but also made with caution: the impaired homeostasis and functions of multiple organs due to aging and comorbidity can contribute to enhance the risk for drug-related adverse events (AEs). Even if adjusted for age and comorbidity score, AD patients consume a higher number of drugs (more anticholinergics, sedatives and antidepressants) [9]: all of which can increase the potential occurrence of side effects due to drug interactions and lead to a cascade of AEs. An Italian group recently reviewed AEs in the placebo arm of donepezil trials, showing that AD patients have a higher risk of developing AEs than people affected by mild cognitive impairment (MCI). This phenomenon may be called “nocebo effect” [17]. In addition, prescribing AD treatments requires caution since almost all safety and tolerability studies to date have been performed within clinical trial settings, where patient’s inclusion criteria were restricted by low comorbidity and/or polytreatment index. In clinical practice, namely the real-world setting, the safety and tolerability profile of AD therapies may be different from that proposed in such studies. Moreover, to date most studies are short term (i.e., 24 weeks): thus, the long-term impact of these medications remains to be determined.

## Aim of the study

The aim of the present study was to focus on the evaluation of the risk–benefit profile of pharmacological therapy for Alzheimer’s disease in relation to somatic comorbidities that often affect these patients. For this purpose a Medline search (from 1998 to 2012) was performed using as key words dementia, Alzheimer’s disease, drug treatment, somatic comorbidities, side effects/adverse events, elderly.

## Current clinical practice: cholinesterase inhibitors

ChEIs are widely recommended as standard therapy in mild to moderate AD by the most important neurological societies. Donepezil, galantamine and rivastigmine possibly decrease Aβ amyloid production and Aβ-induced toxicity, modulate expression of ChE isoforms and increase expression of nicotinic receptors [18]. Some recent studies have demonstrated that donepezil is also effective in severe AD, particularly on the improvement or stabilization of cognitive functions, on global functionality and, although controversial in literature, on behavioral symptoms [19]. Among the other ChEIs, galantamine improved only cognitive function in severe AD, failing to significantly ameliorate functionality in daily living activities [20]. A trial to evaluate safety and efficacy of a higher dose of rivastigmine transdermal patch in severe Alzheimer’s disease is ongoing [21]. Recently the FDA approved use of donepezil in severe AD although data on lifetime cost utility analysis and duration treatment are limited [22]. Interestingly, many randomized controlled clinical trials demonstrated improvement or stabilization of cognitive symptoms as in the severe impairment battery (SIB) and global measures such as the clinical dementia rating (CDR) scale, but the effects on other functional outcomes or behavioral symptoms are inconsistent [22].

The beneficial effects of ChEIs seem to be dose dependent. The use of donepezil at 23 mg daily was recently approved by the Food and Drug Administration in moderate to severe AD, because at this dosage it provided beneficial effects without any additional safety issues [23]. These findings are consistent with previous data: donepezil, because of its ability to bind to serum albumin [24] provided better cognitive improvement in patients with lower serum albumin levels, probably due to a higher drug bioavailability [25].

Even if it is widely known that these agents may improve quality of care and reduce caregiver burden, there is no evidence that they prolong life [26]. The withdrawal of ChEIs can be followed in some patients by worsening of daily functions, so the time to stop treatment must be individualized. However, it seems reasonable to consider not to prolong treatment once a subject has lost the ability to self-care and/or to interact with other people [22].

## Current clinical practice: memantine

Memantine is an adamantine derivative and a noncompetitive glutamate *N*-methyl-d-aspartate (NMDA) receptor blocker. It is the most recent drug to be effective in reducing the rate of cognitive and functional decline in moderate to severe AD [27] and its use has been approved for these stages. It may provide measurable symptomatic effects, but there is at present no clear evidence of benefit in starting memantine at an earlier stage of AD [27]. It has been demonstrated that memantine also improves behavioral symptoms, particularly in the affect, physical behavior and psychosis domains [28] A recent paper by Ballard and co-workers [29] has shown that memantine is at least as efficacious as antipsychotic treatment for the long-term prophylaxis of neuropsychiatric symptoms in people with AD. It seems to have some efficacy also on aphasia, especially on non-fluent subtypes [30]. Memantine is generally prescribed as a second line monotherapy, but its use must be considered as an add-on to ChEIs, even if controversial in literature. When patients with moderately severe Alzheimer’s disease fail on ChEIs, the addition of memantine to rivastigmine may be beneficial without any apparent safety concerns [31]. Tariot et al. [32] have shown benefits from the combination of memantine and donepezil versus donepezil plus placebo on all measures. A more recent study on donepezil and memantine for moderate to severe AD failed to show significant additional benefits of the combinations of the two medications over donepezil alone [33]. On the other hand, another observational study revealed that the addition of memantine to ChEIs significantly altered the history of AD by extending time to nursing home admission [34]. Short- and long-term safety and efficacy profiles for donepezil and memantine have been demonstrated in well-designed trials in post-stroke aphasia. However, it remains to be established whether the concomitant use of these two agents in patients receiving intensive aphasia rehabilitation will provide additional benefits on aphasia recovery [35].

Memantine preferentially blocks excessive NMDA receptor activity that may underlie the degeneration of cholinergic cells, without disrupting its normal activity. As an open-channel blocker, memantine preferentially enters the receptor-associated ion channel when it is excessively open. Most important, its off-rate is relatively fast, avoiding drug accumulation in the channel and preventing interference with normal synaptic transmission [36]. Furthermore, memantine exerts some of its beneficial effects by reducing Aβ amyloid production via blocking calcium entry into neurons. Interestingly, other agents have similar activity: calcium channel blockers (CCBs), commonly used for the treatment of hypertension, target voltage-gated calcium channels on brain neurons in areas involved in both learning and memory. Therefore, the effects of CCBs on both intracellular calcium metabolism and blood pressure regulation can partly explain previous suggestions about the reduction of the incidence and progression of AD by antihypertensive therapies [37].

The efficacy of memantine, probably due to its complex actions, is not only restricted to AD. In patients with mild to moderate vascular dementia, memantine (20 mg/day) consistently improved cognition across different cognitive scales, with no deterioration in global functioning and behavior and no side effects [38]. Chronic administration of memantine in a rat model of diabetic neuropathic pain has also shown antinociceptive effects [39]. Together with ketamine, another NMDA receptor antagonist, it also has a role in tumor-related or in neuropathic pain refractory to opioids [40]. Furthermore, a combined treatment of pregabalin and memantine can decrease pain and the rate of gray matter atrophy associated with fibromyalgia syndrome [41].

Memantine, due to its action on NMDA receptors and its possible neuroprotective effects, has also been studied as treatment for glaucomatous neurodegeneration. Increasing evidences shows a strong link between neurodegeneration in the central nervous system and glaucomatous neurodegeneration by basic cellular processes common to glaucoma and Alzheimer’s disease [42].

## AD patients and somatic comorbidities: main side effects, cautions and warnings to be taken when using ChEIs and/or memantine

ChEIs, because of their cholinergic activity, produce mainly gastrointestinal (GI) and cardiovascular (CV) side effects, but serious AEs are rare. Patients must be carefully monitored for the possible onset of AEs, which are often dose dependent. Dose reduction or withdrawal may be needed.

Common (1–10 %) or very common side effects (>10 %) are nausea, diarrhea, vomiting, abdominal pain/disturbance, fatigue, dizziness and headache. Agitation is common with donepezil and rivastigmine, rare with galantamine. The incidence of GI symptoms, higher in women, is lower for donepezil compared with galantamine and rivastigmine. Rivastigmine GI-related AEs persist during long-term treatment, although its transdermal patch formulation does not show any very common side effects. GI adverse events are the main cause of discontinuation therapy in ChEIs and are mostly due to nausea. Some years ago we described a case of melena following the use of rivastigmine tablets in an elderly man with a history of peptic ulcer [43]. Non-GI AEs have low frequencies and a similar incidence with the different ChEIs.

GI-related AEs are the most common but also the less dangerous. On the contrary, vagotonic effects of ChEIs and the consequent cardiovascular side effects, recommend warnings for their use in patients with many cardiovascular comorbidities such as sick sinus syndrome, sinus-atrial or atrio-ventricular blocks, myocardial infarction, unstable angina or congestive heart failure. Moreover, demented patients taking ChEIs, even in the absence of cardiovascular diseases and with a normal pre-treatment ECG, have a greater risk of developing hypotension, hypertension, atrial fibrillation or, more rarely, bradycardia. Bradycardia is dose dependent for donepezil; it consequently may increase the risk of falls, of having syncope or causing pacemaker implantation, also enhancing the risk of hospitalization due to bone fractures [44]. A meta-analysis of 54 placebo-controlled randomized trials of ChEIs and memantine showed that ChEIs might increase the risk of syncope. Both ChEIs and memantine were found to have little effect on falls, fractures or accidental injuries, even if this review does not exclude a potentially significant risk to underreporting and small number of outcome events [45]. However, a retrospective case note study of 134 patients who started ChEIs therapy for the first time over a 6-month period showed that cardiovascular side effects were rarely a reason for discontinuation of the medication and ECGs were not a useful risk reduction tool to identify cardiovascular side effects in individuals [46]. Of the 21 individuals where the medication had to be stopped, only two patients had cardiovascular problems. The most common ECG abnormalities detected, respectively, were first degree heart block, ventricular ectopics, atrial fibrillation, right and left bundle branch blocks, nonspecific T wave abnormalities and atrial ectopics [46].

However, it is important to identify higher-risk patients; a large study in a real-world setting with a median length of follow-up of over 2 years, showed a greater risk of bradycardia in patients treated with ChEIs, particularly those taking 15 or 20 mg/day of donepezil [47]. A greater risk of low heart rate was shown in patients with dementia diagnosed as non specific or AD, those taking β-blockers, those who had fallen since diagnosis and those with a clinical history of myocardial infarction, heart failure, or hypertension [47].

Caution must be used also for patients with pulmonary diseases (asthma, obstructive pulmonary disease), urinary outflow obstruction, seizures, peptic ulcers and severe hepatic impairment. However, in a population-based, cohort study in patients over the age of 66 years who had concomitant dementia and chronic obstructive pulmonary disease (COPD), new users of ChEIs were not at significantly higher risk of emergency room (ER) visits or hospitalizations for COPD [48]. Among different ChEIs, donepezil and rivastigmine are safe in patients with moderately to severe impaired renal function, while galantamine is contraindicated in severe renal and/or hepatic impairment due to lack of evidence [49, 50]. More rarely, rivastigmine may cause REM sleep behavior disorder (RBD) [51], although there are many literature data on positive effects of ChEIs on sleep. In elderly healthy people, donepezil induces REM sleep augmentation, enhancing memory performances [52, 53]. In autistic patients donepezil can increase the amount of the REM sleep state, ameliorating learning, cognition and behavior [54]. Donepezil is also effective in obstructive sleep apnea syndrome and in narcolepsy, reducing chronic excessive sleepiness [55, 56] and can markedly improve nocturnal symptoms of RBD [57]. A transgenic mouse model of Alzheimer’s disease showed sleep and circadian abnormalities linked to cholinergic transmission [58]. In fact, donepezil can affect REM sleep also in AD patients, enhancing REM sleep and reducing slow frequencies of REM sleep EEG [59]. Rivastigmine can increase REM sleep in the elderly, especially in subjects with significantly reduced REM latency [60]. It ameliorates refractory RBD in patients affected by Parkinson’s disease [61]. In Lewy body dementia, rivastigmine produced significant clinical improvement in patients with sleep disruption and in a case report also immediately resolved agrypnia and nocturnal confusional behaviors [62, 63]. Galantamine is the less studied ChEI for its effects on sleep. However, the relationship between the beneficial effects of ChEIs on REM sleep and its efficacy on cognitive decline remain still unclear.

More rarely, ChEIs may cause the occurrence of movement disorders (rivastigmine-induced dystonia and Pisa syndrome, donepezil-induced chorea) [64–66] and toxic hepatitis with or without cholestasis (rivastigmine, donepezil) [67, 68].

There is at present no available prescribing information in severe AD, but literature data indicate that it is similar in different AD stages.

Another crucial point is related to the concomitant use of ChEIs and bladder anticholinergics, such as oxybutynin or tolterodine. In higher-functioning nursing home residents, dual use of ChEIs and bladder anticholinergics may result in greater rates of functional decline than use of ChEIs alone. The minimum data set (MDS)-cognition scale (COGS) used, in this study, may not be sensitive enough to detect differences in cognition due to dual use [69].

Among ChEIs, donepezil has a clear affinity to serum albumin. Therefore, conditions that cause hypoalbuminemia (including cirrhosis, malnutrition, nephrotic syndrome and sepsis) can increase the bioavailability of donepezil and consequently the risk of AEs. It is reasonable to monitor such patients more strictly.

Memantine seems to have a better profile of safety and tolerability: AEs are very low and similar to placebo and very common symptoms are not reported. The most common AEs are constipation, headache, hypertension and somnolence. Furthermore, memantine can reduce or prevent agitation/aggression and has a lower withdrawal compared to placebo [70]. On the other hand, there are case reports describing memantine-induced heart failure and hepatitis without cholestasis [71, 72]. Warnings for its use concern the presence of epilepsy, raised urinary pH, myocardial infarction, uncompensated congestive heart failure, uncontrolled hypertension and severe hepatic impairment [70].

**Fig. 2:**
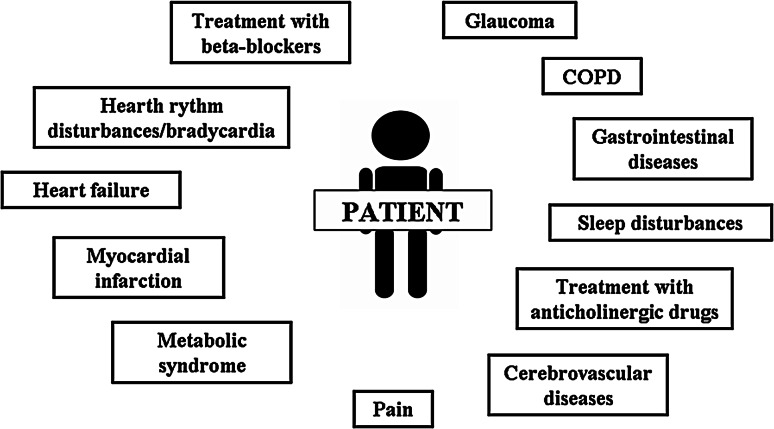
Complexity in the treatment of a demented patient with comorbidities

Figure 2 reports the main points to be taken into account when a demented patient with comorbidities has to start treatment.

## A common threat: drug interactions

A common threat for AD treatment in presence of comorbidity is due to drug interactions. The risk for interactions increases with age, number of diseases and number of drugs used; this is usually a factor in elderly people [73].

In terms of drug interactions, ChEIs enhance the effects of succinylcholine-type muscle relaxant action during anesthesia. Their common concomitant use with bladder anticholinergics for urinary incontinence in demented patients occurs in one-third of patients and causes a greater functional decline than when used alone. There are no data about the efficacy of ChEIs associated with anticholinergic agents for other frequent conditions (i.e., depression). Galantamine may interact with digoxin and beta-blockers, reducing heart rate. Levels of donepezil and galantamine are modified by inhibitors (such as paroxetine or fluoxetine) and by enhancers (such as rifampicin, alcohol or phenytoin) of CYP3A4 and CYP2D6 [70, 73] (Table 1). ChEIs do not interfere with warfarin or digoxin. Also donepezil, although showing affinity to serum albumin, does not alter warfarin levels [74].

**Table 1:** Some examples on interactions among ChEIs, memantine and other drugs via CYP450

Cytochrome	Substrates	Inhibitors	Inductors
CYP3A4	Antipsychotics: haloperidol, clozapine, risperidone, ziprasidone, sertindole, quetiapine, aripiprazole Antidepressants: tricyclicics, venlafaxine, citalopram, mirtazapine Benzodiazepines: diazepam, bromazepam Nonbenzodiazepine anxiolytics: buspirone Anticonvulsants: carbamazepine, felbamate, tiagabine Calcium antagonists: nifedipine, diltiazem, verapamil Other drugs: macrolides (erythromycin, clarithromycin), terfenadine, astemizole, tamoxifen, cyclosporine, amiodarone, quinidine AChEIs: donepezil, galantamine^a^	Antifungal drugs (ketoconazole, fluconazole, itraconazole) Erythromycin Fluvoxamine Nefazodone Grapefruit juice (at least 250 ml)	Barbiturates Phenytoin Rifampicin Carbamazepine Hypericum Topiramate^b^ Oxcarbazepine^b^ Felbamate^b^
CYP2D6	Antipsychotics: haloperidol, thioridazine, perphenazine, fluphenazine, zuclopentixol, risperidone, clozapine, olanzapine, aripiprazole Antidepressants: tricyclicics, fluvoxamine, fluoxetine, paroxetine, citalopram, mianserine, venlafaxine, mirtazapine Opiates: codeine, tramadole, dextrometorphan β-Blockers: metoprolol, propranolol, pindolol, timolol Antiarrythmics: propaphenon, flecainide, encainide Other drugs: debrisoquine, spartein, phenformin AChEIs: donepezil, galantamine^a^	Fluoxetine Paroxetine Quinidine Propaphenon Thioridazine Perphenazine	No known agent

^a^Neither the AChEI rivastigmine nor the NMDA receptor antagonist memantine present hepatic metabolism. Protein binding is 96 % for donepezil, 40 % for rivastigmine, 18 % for galantamine and 145 % for memantine

^b^Weak enzymatic inductor

Among its interactions with other drugs, memantine can enhance anti-parkinson and anticholinergic agents, by reducing the effects of barbiturates and neuroleptics (Table 1). Memantine is contraindicated in concomitant use with amantadine, ketamine or other agents acting on the same NMDA receptor; it interacts negatively with phenytoin, reduces hydroclorothiazide levels and may increase warfarin levels [70].

## Conclusions

Occurrence of age-related comorbidities is a crucial concern in the choice of treatment in Alzheimer’s disease. Somatic diseases may worsen cognitive and/or behavioral symptoms in AD patients, already described in literature as sicker, frailer and more susceptible to pharmacological AEs than the older population without dementia.

Cholinesterase inhibitors and memantine are the principal agents used in the management of Alzheimer’s disease. Only after a careful evaluation of comorbidities can clinicians proceed to select a specific AD treatment, taking into account the safety and tolerability of different drugs. This selection is important in order to maximize drug efficacy and optimize quality of life, avoiding adverse reactions that can complicate clinical course [75].

Numerous conditions common in Alzheimer’s disease, particularly cardiovascular diseases, may restrict use and/or efficacy of ChEIs. They can also interact with many drugs frequently prescribed in the elderly, especially in AD patients (anticholinergic agents, paroxetine, fluoxetine). Not age per se but the overall clinical condition of a patient affected by Alzheimer’s disease, including care dependency and geriatric comorbidities, influences the process of decision making on ChEIs prescription. Patients with more chronic conditions and polytreatment received a prescription for ChEIs less frequently [16]. Conversely, we must consider the benefits of a specific treatment for dementia that can decrease disease severity and the consequent risk for long-term care. Compared to ChEIs, memantine seems to have a good profile of efficacy and tolerability with a better safety in pulmonary, cardiovascular and central nervous system comorbidities. Memantine’s drug interactions are also more favorable since they mostly concern drugs not commonly used in older people. Moreover, given its effects on behavioral symptoms and language disturbances, memantine may be useful in AD subtypes overlapping with frontotemporal lobar degeneration.

Clinicians usually prescribe treatment with ChEIs when patients present low comorbidities, starting treatment with memantine when patients have medium–high somatic burden. The influence of the severity of comorbidities on the drug choice underlines the importance of caution taking into account the somatic dimension in AD management. When ChEIs are not tolerated there is a common consensus on the opportunity of stopping the drug and prescribing memantine. However, there is no common consensus in stopping the ChEIs or reducing their dosage and re-assessing the patient (Bianchetti et al. 2011, personal communication).

In conclusion, the treatment of the AD patients with somatic comorbidities requires a weighted and complex approach which includes an accurate assessment and staging of the associated diseases, the complete evaluation of all pharmacological treatments, the focus on “his/her” Alzheimer’s disease, the potential benefits from a specific therapy, and the careful evaluation of AEs which can potentially arise in that patient. Overall, these concerns underline the need for individual management as an effective strategy to ameliorate the quality of life in Alzheimer’s disease patients [73].
